# Habitat Use of Birds on a High‐Mounted Agrivoltaic Trial Plot

**DOI:** 10.1002/ece3.71864

**Published:** 2025-08-14

**Authors:** Lara Diekmann, Felix Zitzmann, Frank Schaarschmidt, Michael Reich

**Affiliations:** ^1^ Institute of Environmental Planning Leibniz University Hannover Hannover Germany; ^2^ Institute of Cell Biology and Biophysics, Biostatistics Section, Leibniz University Hannover Hannover Germany

**Keywords:** avifauna, biodiversity, camera traps, photovoltaic, renewable energies

## Abstract

Agrivoltaic is the dual use of land for agriculture and solar energy generation and can therefore be an opportunity to prevent land‐use conflicts with food production in the context of renewable energy expansion. At the same time, agrivoltaic could have an impact on biodiversity, especially on farmland birds. We investigated the habitat use of birds on a high‐mounted agrivoltaic trial plot with chive culture ( *Allium schoenoprasum* ) in northern Germany. Over a one‐year period, we surveyed bird use on the *agrivoltaic* plot, in three neighboring plots (without agrivoltaic but also cultivated with chives of different age‐classes) and an intervening tree row at two‐week intervals using standardized bird counts. Additionally, we detected bird use within the *agrivoltaic* plot and an adjacent *control* plot by camera traps. The results of the bird counts showed no significant differences in the number of species per visit between the *agrivoltaic* and the other chive plots (except the tree row) as well as in the number of observations per visit between *agrivoltaic* and any of the other plots. However, the direct comparison of *agrivoltaic* and *control* showed that the number of species and observations per visit were higher on the *agrivoltaic*. Camera trapping revealed no difference between *agrivoltaic* and *control* in terms of the number of bird species and detections per camera trap. Most of the species with high presence on the agrivoltaic were widespread, common species, adapted to anthropogenic structures, like White Wagtail and Black Redstart. In contrast, the Yellow Wagtail, a species of open landscapes, showed a higher visit frequency and use intensity on the *control*. Overall, the investigated agrivoltaic did not appear to prevent habitat use of most farmland birds—apart from bird species of open landscapes. However, there is a considerable need for further research on the effects of agrivoltaic on birds and biodiversity.

## Graphical abstract

Over a one‐year period, we surveyed bird use on a high‐mounted agrivoltaic trial plot, in three neighboring plots (without agrivoltaic but with same crop of different age‐classes) and an intervening tree row at two‐week intervals using standardized point counts. In addition, we detected bird use within the agrivoltaic plot and an adjacent control plot by camera traps.

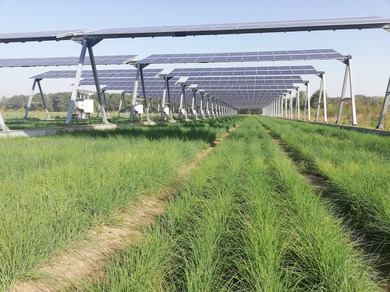

## 1 Introduction

The challenges of climate change and the need for decarbonization as well as the increasing global demand for energy induced a global energy transition (IPCC 2023; Steinberg 1999). Renewable energy sources are expected to generate more than one‐third of the world's electricity in 2025, which is largely supported by the expansion of photovoltaics (IEA (International Energy Agency) 2024). Solar energy is the most widely available energy source on earth, and its increasing utilization is being pushed by technological trajectories and political implementations (Nijsse et al. 2023; Colasante et al. 2022). Therefore, a stronger competition for land is expected, especially with food production (Pascaris et al. 2021; van de Ven et al. 2021; Widmer et al. 2024). To face this challenge, agrivoltaic systems could be a helpful approach as they combine solar energy generation and agricultural use on the same area (Dupraz et al. 2011; Weselek et al. 2019). In addition, this technology could be an advantage for agriculture in the course of climate change, as shading of the solar panels can reduce evaporation and increase water availability during droughts (Marrou et al. 2013; Elamri et al. 2018; Adeh et al. 2018).

At the same time, the implementation of agrivoltaic systems leads to changes in the landscape, having visual impacts and causing changes regarding land use, cultivated crops, relief, and openness (Ketzer et al. 2020; Sirnik et al. 2024; Widmer et al. 2024). As a result, effects on biodiversity are to be expected, but these have hardly been investigated to date (Gómez‐Catasús et al. 2024). Having the greatest potential on arable land and grassland (Adeh et al. 2019), photovoltaic power plants, including agrivoltaic systems, will affect the biodiversity of agricultural landscapes and could be challenging, especially for bird species, since breeding populations of common farmland birds in Europe are continuously declining (Donald et al. 2001; Reif et al. 2022).

There are some evaluations of the environmental impacts and the effects of ground‐mounted solar parks on various species groups, including birds (DeVault et al. 2014; Montag et al. 2016; Jarčuška et al. 2024; Golawski et al. 2025). Some studies show that areas covered with solar panels are barely used for breeding, and especially birds of open landscapes may avoid these areas (Gabriel et al. 2018; Golawski et al. 2025; Hemmer et al. 2025). On the other hand, solar parks can be attractive for foraging birds (Montag et al. 2016; Herden et al. 2009) and some species seem to use the sub‐construction of the solar panels for breeding (Jarčuška et al. 2024). Some studies even found a higher diversity and species richness of birds within solar parks than on arable land or intensive grassland control plots (Montag et al. 2016; Jarčuška et al. 2024), which may also be related to newly created vegetation structures surrounding these areas, such as hedges (Gabriel et al. 2018). Many of the findings show that ground‐mounted solar parks can have positive effects on bird species, especially in intensively used agricultural landscapes, as in solar parks, usually grasslands develop that are extensively managed without the use of fertilizers and pesticides (Golawski et al. 2025). However, these characteristics do not apply for agrivoltaic systems, where the focus is still on agricultural use (Dinesh and Pearce 2016). Land‐use intensity is an important factor for the habitat use by birds (Verhulst et al. 2004; Li et al. 2023) and in addition, the design of agrivoltaic systems (sub‐construction, system height) is different from that of ground‐mounted systems and may, therefore, have different effects on birds. Findings on the effects of ground‐mounted solar parks on bird species are thus not transferable to agrivoltaic systems. These effects are so far unknown.

As a first step to closing this knowledge gap, we investigated the habitat use of (farmland) birds on a high‐mounted agrivoltaic trial plot and in adjacent control plots with the same crops over a one‐year period. Our case study is of descriptive and preliminary nature, shows first results on habitat use (but not breeding behavior) by birds on a small‐scale agrivoltaic, and addresses the following research questions: (1) Which bird species use the agrivoltaic and surrounding plots as habitat and how frequently do they occur? (2) Are there differences regarding the number of species and their habitat use between *agrivoltaic* and a directly adjacent *control* plot?

## 2 Material and Methods

### 2.1 Study Area and Sample Plots

**FIGURE 1:**
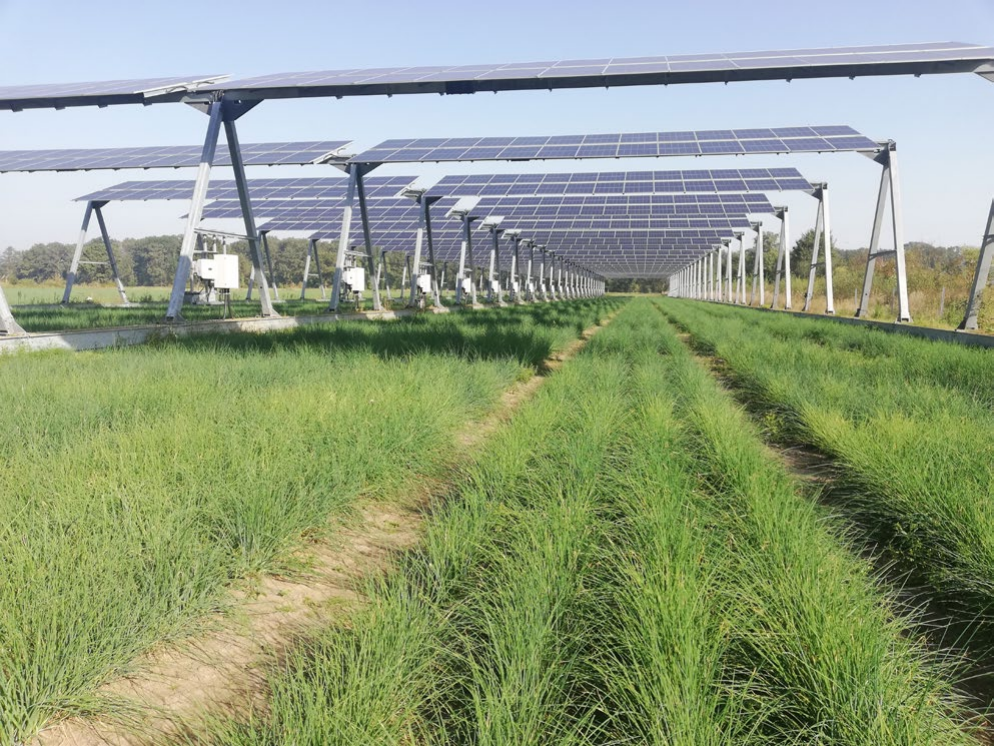
The high‐mounted agrivoltaic trial plot is a south‐facing system with 24 rows of solar panels at a height of approx. 6 m. Chives ( *Allium schoenoprasum* ) are cultivated under it with each management strip comprising four rows of chives. Photo taken by F. Zitzmann on 11 September 2023.

**FIGURE 2:**
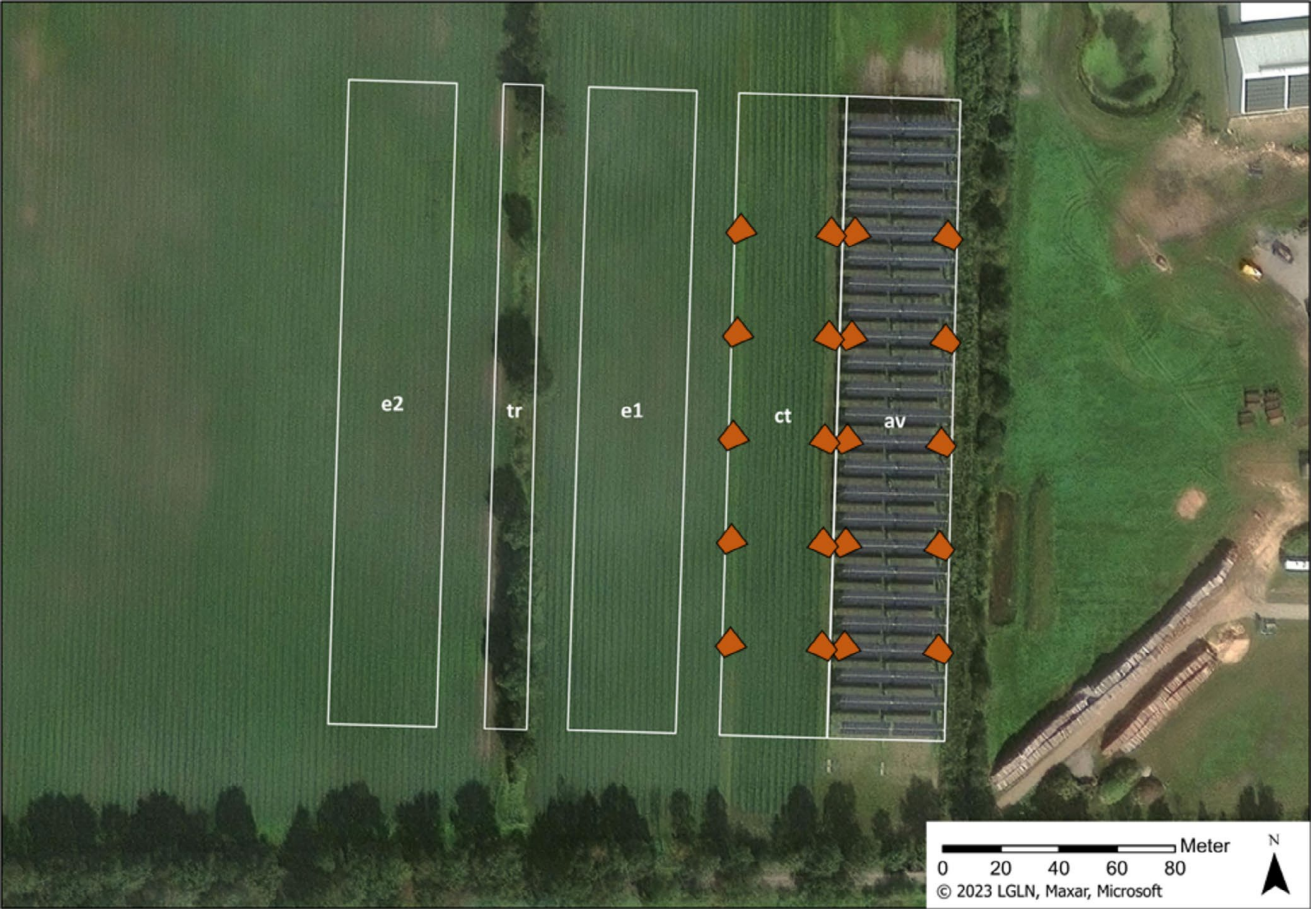
View on the sample plots bordered in white: av = agrivoltaic, ct = control (both sown in 2022), e1 = established I (sown in 2021), e2 = established II (sown in 2018), tr = tree row. All sample plots apart from the tree row have a size of 37 × 220 m (approx. 0.8 ha). The camera traps (shown in orange; *n* = 20) were positioned on agrivoltaic and control and detected both plots from east and west with direction slightly to the north.

We conducted our research on an agrivoltaic trial plot built in 2022 in the northeast of Lower Saxony (district Lüchow‐Dannenberg, Northern Germany, 53°00′13.3″N 11°10′27.3″ E). The surrounding area is characterized by agriculture (arable land and grassland), with a forest in the north and structured by hedges and tree rows. The agrivoltaic is a high‐mounted, south‐facing system with 24 rows of solar panels at a height of approx. 6 m (Figure 1) and a size of 0.8 ha. Under the solar panels, chives ( *Allium schoenoprasum* ) were cultivated as a perennial crop, meaning intensive farming with frequent harvests (about every 20 days) and maintenance (fertilizer or pesticide use) leading to a disturbance every 9.3 days (SD 8.4) during the vegetation period. We investigated the bird use on five sample plots, including the *agrivoltaic* plot itself and an adjacent *control* plot (both sown with chives in September 2022) as well as two plots with older chives sown in 2021 (*established I*) and 2018 (*established II*). The size of each plot was based on the size of the agrivoltaic system (37 m x 220 m). In addition, we investigated a *tree row* located in the area, representing a natural vertical element similar to the height of the high‐mounted agrivoltaic system (Figure 2).

### 2.2 Bird Surveys by Standardized Counts

We surveyed the presence of birds on 27 days in a two‐week interval (8 November 2022 to 6 November 2023) on all five sample plots shown in Figure 2. Our approach was inspired by point counts (Bibby et al. 2000), but strongly modified. Each of the plots (except the *tree row*) included six management strips running in a north–south direction, with one management strip comprising four rows of chives (see Figure 1). For our surveys, we first walked by the south side of the plots and stopped at each management strip for an observation time of 1 min (=6 min observation time per plot). Prior to observation, a short settling period (1 min) was implemented (to reduce disturbance and allow birds to resume to normal behavior) at each plot. In this way, we observed all six management strips of each plot one after the other. We then repeated the same procedure on the north side of the plots. Thus, on each of the 27 survey days, every plot was observed for 12 min in total (6 min from south‐ and 6 min from north‐side). The tree row was observed by walking by both sides (east and west) for 6 min each (=12 min observation time per survey day). All birds observed visually or acoustically on the plots were noted with the species name and the maximum number of individuals present at the same time. Birds flying over were not recorded. Surveys usually started at 10 a.m. and were only carried out under good weather conditions (no rain, no strong wind).

### 2.3 Detection of Birds with Camera Traps

To supplement our standardized counts, we used time‐triggered camera traps (CTs) since this method combination can be beneficial to record bird communities in areas with low bird densities/activity more reliably (Wix and Reich 2019). We used the CTs only on the *agrivoltaic* and the *control* plot, as these two plots had the same characteristics (age, crop, management), apart from the solar panels of the *agrivoltaic*. The survey was carried out with 20 CTs (Dörr Snapshot Mini Black). These were positioned so that both plots were monitored from east and west, each with 5 evenly distributed CTs per side (=10 CTs per plot) which were placed at a distance of approx. 40 m from each other in a row (Figure 2). The CTs were attached at a height of 100 cm with a slight downward angle to detect birds in the close‐up range, facilitating the detection of even small species. The CTs worked time‐triggered and took pictures in an interval of 10 min (=144 pictures per camera and day). They were active continuously from 8 November 2022 to 6 November 2023 (363 days) and ran the exact same time (without failures).

### 2.4 Data Analysis

All statistical analyses were performed using R, version 4.4.0 (R Core Team 2024). When analyzing the data of the bird surveys by standardized counts, the maximum number of observations per species within the 12‐min observation time was considered for each sample plot. The data of the individual dates from the five plots were regarded as related samples (same date/season, weather and basic conditions). To test for differences in habitat use (number of species and detections per visit) between the five sample plots, we performed a Friedman Test for related samples using the R Package “psych” (Revelle 2024). Afterwards, we performed a Friedman Post hoc‐Test for multiple comparisons between the five sample plots using the “friedmanmc” function in the R package “pgrimess” (Giraudoux 2024). As the comparison *agrivoltaic* and *control* was particularly relevant (as they were next to each other and identical except for the solar panels), we additionally performed a Wilcoxon Test for related samples to directly compare these two plots using the “wilcox.test” function in the R Package “psych”.

To analyze the results of the CTs on *agrivoltaic* and *control*, we aggregated the days with presence and the number of detections for the most common species revealed by all CTs per plot. While the days with presence were used as an indicator for the visit frequency, the number of detections was used as an indicator for the use intensity (Armenteros et al. 2021). One detection represents one CT picture with bird evidence (regardless the number of individuals). To determine differences in habitat use between both plots, we also analyzed the data on CT‐level considering these two parameters: (1) the visit frequency, represented by the number of days with presence per CT and (2) the use intensity, represented by the number of detections per CT. The data analysis of the CTs was performed using the R package bundle “tidyverse” (Wickham et al. 2019) for data preprocessing. We performed the analyses for the number of species per CT as well as for the visit frequency and the use intensity, with the last two both for all species together and for the four most common species detected. Prior to statistical analysis, data were log_10_ transformed to account for data with right skewed distribution and variance increasing with increasing mean. In order to roughly account for spatial dependencies, generalized least square models (gls, R package “nlme” (Pinheiro and Bates 2000)) were fitted to the transformed data: a categorical variable distinguishing *agrivoltaic* vs. *control* was the only predictor in the model; spherical correlation structures without nugget effect were assumed for the model residuals to account for spatial correlations. Based on estimated means, mean differences and standard errors derived from these fitted models, tests for the difference between *agrivoltaic* and *control* were performed on the log_10_ scale and then back transformed (R package “emmeans” (Lenth 2025)). Although the presented statistical analysis attempts to account for the lack of independence due to the spatial vicinity of the CTs, this analysis cannot overcome the lack of independent replications at different environments. As our investigation is a case study, the results cannot be generalized but can provide first insights into the use of agrivoltaic plants by birds. Such investigations should be repeated in further studies with a larger sample size and independent replications.

## 3 Results

We found a total of 45 species (see appendix: Table A1), of which 37 were detected by our standardized bird counts and 24 by CTs. As expected in this area, we mainly recorded species typical for semi‐open agricultural landscapes. By far the most common species was the White Wagtail (recorded on 42% of recording days by observation and/or CTs); other common species were Common Linnet, Yellow Wagtail, and Wood Pigeon (on at least 11% of recording days). Less common (5%–10% of recording days) species were Black Redstart, Yellowhammer, Tree Sparrow, Woodlark, Common Chaffinch, and Common Starling. Five of the detected species are on the Red List for Breeding Birds in Germany (Ryslavy et al. 2020), namely Common Linnet, Common Starling, Eurasian Skylark, Common House Martin (all vulnerable) and Gray Partridge (endangered).

### 3.1 Bird Presence on the Plots Verified by Standardized Counts

Our standardized counts on the five plots resulted in 37 species with a total of 1175 bird observations. Regarding the total number of species and observations per plot (Table 1), the *agrivoltaic* took an intermediate position between *control* and *established I* (less species and observations than *agrivoltaic*) and *established II* and the *tree row* (more species and observations than *agrivoltaic*). With regard to the number of species per visit, the values in the *tree row* were significantly higher than in all other plots, while the four other plots (including *agrivoltaic*) did not differ (Table 1). The number of observations per visit was highest in the *tree row* and in *established II* and lowest in *control*. The *agrivoltaic* took an intermediate position and did not differ from any of the other plots. An additional direct comparison of *agrivoltaic* and *control* using a Wilcoxon test, however, revealed that the numbers of species and observations per visit both were significantly higher on *agrivoltaic* (*p =* 0.007 resp. *p =* 0.002).

**TABLE 1:** Numbers of species and observations in total and per visit (Mean ± SE) on the five sample plots (av = agrivoltaic, ct = control, e1 = established I, e2 = established II, tr = tree row) determined by bird surveys (with a total of 27 visits per plot).

	av	ct	e1	e2	tr	*p*
No. of species						
Total	17	11	12	21	26	
Per visit	2.2 ± 0.4^a^	1.1 ± 0.3^a^	1.6 ± 0.3^a^	2.5 ± 0.5^a^	4.3 ± 0.4^b^	< 0.001
No. of observations						
Total	156	62	146	443	368	
Per visit	5.8 ± 1.3^abcd^	2.3 ± 0.7^b^	5.4 ± 1.5^bc^	16.4 ± 4.9^cd^	13.6 ± 2.8^d^	< 0.001

*Note:* Differences were tested with Friedman test. Multiple comparisons between groups were done using Friedman Post hoc test and different letters indicate significant differences between individual plots (*p* < 0.05).

**FIGURE 3:**
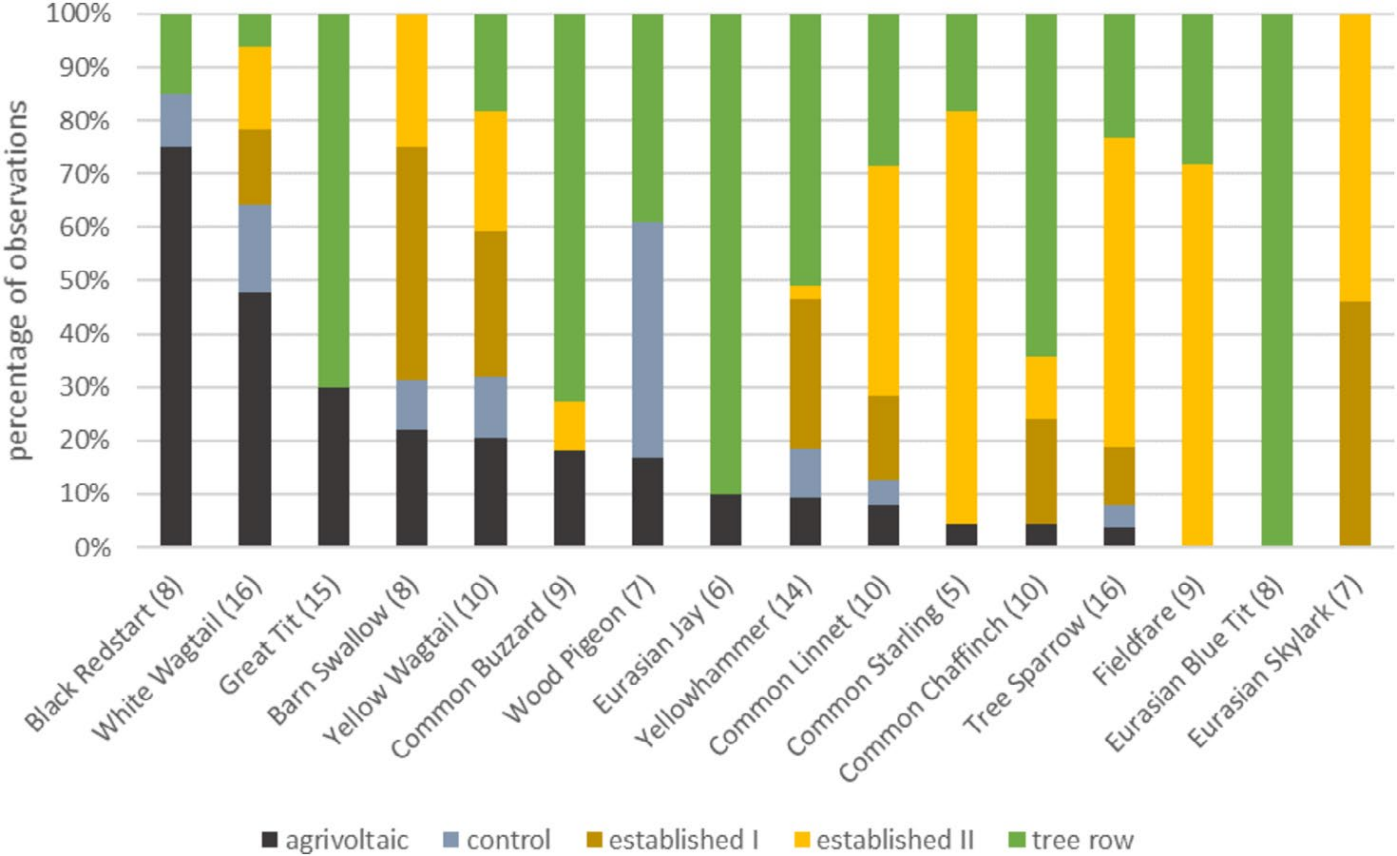
Proportion of observations of the most common species (with observations on at least 20% of the survey days (*n* = 27), total no. of days with observation in brackets) on the individual sample plots, sorted from left to right by highest proportion on agrivoltaic.

The species with the highest percentage of observations on the *agrivoltaic* were Black Redstart (75% on *agrivoltaic*) and White Wagtail (48% on *agrivoltaic*), with Black Redstart only using the plots *agrivoltaic*, *control*, and *tree row*, while White Wagtail was observed on all five plots (Figure 3). Most of the common species (detected on at least one third of survey days) had less than 20% of the observations on the *agrivoltaic* but preferred the *tree row*, *established I*, and *established II*. Some species were not observed at the *agrivoltaic* or the adjacent *control* at all; for instance, Fieldfare was only found on *established II* and *tree row*, Eurasian Blue Tit only at the *tree row*, and Eurasian Skylark only on *established I* and *established II*. Other species like Great Tit, Wood Pigeon, and Eurasian Jay were never surveyed on the established fields but on the *agrivoltaic*, the *control*, and/or the *tree row*.

### 3.2 Habitat Use on *Agrivoltaic* and *Control* Plot Revealed by Camera Traps

**FIGURE 4:**
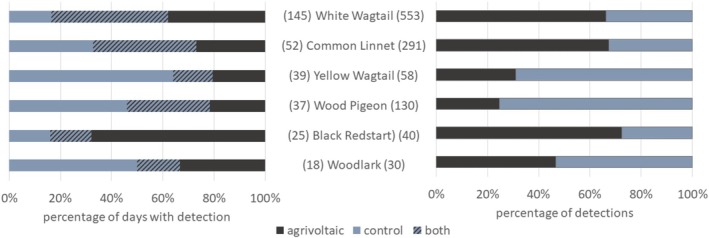
Proportion of days with detection (left) and proportion of detections (right) for the most common species (observed on at least 5% of survey days and with a total of at least 30 detections) on the two sample plots (with *n* = 10 CTs per plot). Species are sorted by their frequency, showing the number of days with detection on the left of the species name (*n* = 363 days investigation period) and the total number of detections on the right of the species name (with a total of 1291 detections during investigation period for all species together).

Overall, most of the detections by CTs were made during the main breeding season (April to July), which applies to both *agrivoltaic* (82% of all detections) and *control* (90% of all detections). The CTs recorded a total of 24 bird species, with more species on the *agrivoltaic* (21) than on the *control* (17). In addition, 60% of the total of 1291 detections were made on the *agrivoltaic* and 40% on the *control*. White Wagtail was by far the most common species during the investigation period (Figure 4), with presence on 40% of the recording days (145 of 363 days) and the highest total number of detections (533). The species was detected on more days and with more detections on the *agrivoltaic* than on the *control*. The same applies to Common Linnet and Black Redstart, which, however, were recorded less frequently than White Wagtail. In contrast, Yellow Wagtail and Wood Pigeon were less active on *agrivoltaic*, with around three quarters of detections on *control* (Figure 4). Woodlark was about equally active on both plots.

Although the mean values for the number of species, visit frequency (no. of days with detection) and use intensity (no. of detections) per CT were all slightly higher on *agrivoltaic* than on *control*, both plots did not differ significantly (Table 2). The species‐specific results for the four most common species (with detection on at least 10% of overall recording days) showed slightly but not significant differences for White Wagtail, Common Linnet, and Wood Pigeon, with the first two using the *agrivoltaic* slightly more actively and the last showing slightly higher activity on the *control*. The only species with a significant difference was the Yellow Wagtail, with a higher use intensity and visit frequency on the *control*.

**TABLE 2:** Mean values (± SE) of measured variables per CT on the agrivoltaic and control plot (with *n* = 10 CTs used per plot) during the investigation period (363 days).

Variable	*Agrivoltaic*	*Control*	*p*
No. of species per CT	9.1 ± 1.0	7.4 ± 0.8	0.21 ^n.s^
Visit frequency^a^	40.1 ± 6.5	32.7 ± 5.3	0.37 ^n.s^
White Wagtail	21.0 ± 3.5	14.3 ± 2.4	0.13 ^n.s^
Common Linnet	8.7 ± 3.1	5.0 ± 1.8	0.28 ^n.s^
Wood Pigeon	0.4 ± 0.4	1.8 ± 1.8	0.06 ^n.s^
Yellow Wagtail	1.3 ± 0.3	**3.1 ± 0.7**	0.01*
Use intensity^b^	66.0 ± 14.1	45.7 ± 9.7	0.23 ^n.s^
White Wagtail	28.0 ± 5.4	16.5 ± 3.1	0.07 ^n.s^
Common Linnet	15.1 ± 6.0	5.7 ± 2.3	0.10 ^n.s^
Wood Pigeon	0.3 ± 0.4	2.3 ± 2.8	0.05 ^n.s^
Yellow Wagtail	1.4 ± 0.3	**3.3 ± 0.8**	0.02*

*Note:* Results shown for all species together and for the four most common species detected by CTs (=species detected in total on at least 10% of the recording days). Differences were tested with GLS‐Models, significantly greater values are marked in bold.

Abbreviation: n.s., not significant.

^a^ No. of days with detection per CT.

^b^ No. of detections per CT.

* Significant *p* < 0.05.

## 4 Discussion

The 10 most common species were recorded on various sample plots and all of them also on the *agrivoltaic*, showing that a complete avoidance by these typical species in this landscape was not recognizable. Some species had even higher activities on the *agrivoltaic* than on the other plots, but other species used the plots without agrivoltaic more actively. The direct comparison between *agrivoltaic* and *control* plot, which had the same age and were managed in exactly the same way, showed more species and detections on the *agrivoltaic* (proven both by standardized counts and CTs). This result is similar to findings of some studies at ground‐mounted solar parks where the numbers of species were higher on solar parks than on arable land or intensive grassland control areas, often explained by the more extensive land use and a higher structural diversity on the solar parks (Montag et al. 2016; Jarčuška et al. 2024; Golawski et al. 2025; Copping et al. 2025). At agrivoltaic facilities, however, the conditions are different (intensive land use, very few habitat structures) and in our study, most of the species with high presence on the *agrivoltaic* are widespread and often adapted to anthropogenic structures, like White Wagtail and Black Redstart. These species did not seem to be disturbed by the technical structures and even used the agrivoltaic as a song post.

Some species of semi‐open agricultural landscapes also showed higher use intensities on the *agrivoltaic* than on the *control*; for instance, Yellowhammer, the vulnerable Common Linnet, and the endangered Gray Partridge. All three favor structured agricultural landscapes with hedges and margins (Andretzke et al. 2005). These structures could be found directly next to the agrivoltaic of our study (e. g. an early succession hedgerow in the east). For Gray Partridge, which we, however, detected only rarely (on 2% of days with detection by CTs during investigation period), it is known that avoidance behavior towards human infrastructure has decreased over the last decades due to generally decreasing habitat quality (Harmange et al. 2019), which may explain the acceptance of the agrivoltaic in its habitat. Additionally, Yellowhammer prefers territories containing suitable song posts (McHugh et al. 2016), which were offered by the high‐mounted *agrivoltaic*. In fact, we observed not only Yellowhammer but also, for example, White Wagtail, Black Redstart, and especially Yellow Wagtail sitting and/or singing on the solar panels. Remarkably, the only significant difference between *agrivoltaic* and *control* occurred for Yellow Wagtail, which had significantly more days with detection and higher detection numbers per CT on the *control*. This species prefers open landscapes but also needs high song posts (Andretzke et al. 2005). This probably explains the higher use intensity on the field level of the *control*, while the *agrivoltaic* was mainly used as a song post. This can be confirmed by our standardized counts, which showed similar proportions of observations of Yellow Wagtails on *agrivoltaic*, *established I*, and *established II*, but most of the observations on *agrivoltaic* were made by individuals sitting on the solar panels. Overall, its preference for the more open *established* plots can be recognized.

Another species of open landscapes we detected in the study area is the Eurasian Skylark, which, unlike the Yellow Wagtail, needs relatively large open fields and keeps a distance to vertical structures (Rahman et al. 2012; Wagner et al. 2014). The populations of Eurasian Skylark strongly declined over the last decades (Tirozzi et al. 2024) and the species is declared vulnerable in many European countries (e. g. in Germany; Ryslavy et al. 2020). We detected Eurasian Skylarks mainly on the plots *established I* and *established II*, but also on the *control*, where, however, it was detected (by CTs) at the very edge, with the greatest possible distance from the agrivoltaic (approx. 40 m) and only rarely (< 1% of days with detection by CTs during investigation period). On the *agrivoltaic*, however, we did not detect Eurasian Skylarks at all, neither through our standardized counts nor through the CTs. Since the study area was part of a semi‐open and structured landscape, it may by itself not be an optimal habitat for the Eurasian Skylark (probably resulting in low population densities there). Studies at ground‐mounted solar parks (however, with focus on breeding birds) detected Eurasian Skylarks only in larger open areas (e. g. by larger module row spacings) or at the edges of the plants (Montag et al. 2016; Tröltzsch and Neuling 2013) and proved lower abundances within the solar parks than on control sites with grassland or arable land (Jarčuška et al. 2024; Golawski et al. 2025). In a study of 30 solar parks in Southern Germany, Hemmer et al. (2025) observed only rare use by Eurasian Skylark (as song post or for foraging); however, breeding of Eurasian Skylarks was detected at none of the solar parks but only at control sites outside the facilities. The avoidance of high‐mounted agrivoltaic systems by Eurasian Skylark could be even stronger due to their greater height compared to ground‐mounted solar parks.

We want to emphasize that the habitat use and activity of the bird species detected in our study do not allow any conclusions to be drawn on the use of the area for breeding, as our methods were not designed for this purpose. Niche breeders like White Wagtail or Black Redstart, which we observed regularly (also with juveniles of White Wagtail), are most likely to breed at the sub‐construction of agrivoltaic facilities as this has already been confirmed on ground‐mounted solar parks (Jarčuška et al. 2024). Beyond that, mainly the edge areas of solar parks are used for breeding while the areas with solar panels are rather used for foraging (Gabriel et al. 2018). This also seems to apply to high‐mounted agrivoltaic and may even be more pronounced there, as the focus of these facilities is on (economic) agricultural use (Dupraz et al. 2011; Dinesh and Pearce 2016). Therefore, the land use is more intense than at ground‐mounted solar parks. For instance, management measures and harvesting of the chives at the *agrivoltaic* facility studied here led to frequent disturbance of the area from mid‐April to the beginning of September, so that no ground‐nesting bird species would have been able to complete a successful breeding (cf. Andretzke et al. 2005). However, the area was still used for foraging, especially during the breeding season, as the concentration of detections by CTs in the months April to July indicates.

Overall, the *agrivoltaic* trial plot investigated here did not appear to be an obstacle to habitat use for most bird species—apart from those of open landscapes. However, as we did not perform a before‐after comparison, it is possible that species have already been displaced from the area by the installation of the facility. Therefore, although our study provides important initial results, it cannot fully evaluate the effects of this *agrivoltaic* on birds. Furthermore, due to their spatial proximity, all sample plots were still influenced by the *agrivoltaic* (e.g., by visual impacts) and differences in habitat use between them could also be explained by their distance to the *agrivoltaic*, adjacent structures (e.g., tree rows and hedges in the area) or the different ages of the chives on the plots (although the frequent maintenance and harvesting on all plots lead to hardly any structural differences between different age‐classes of chive). Also, due to the lack of plot independence, potential pseudo‐replication and site‐specific context, our findings are non‐generalizable but must be seen as initial insights. Finally, it must be emphasized that our study was carried out at a single small‐scale *agrivoltaic* and the results could be different at larger facilities and in different landscape contexts. Looking at the current development, the number of installed agrivoltaic facilities and their size will strongly increase in the next years (Walston et al. 2022; Pump et al. 2024). Further research is needed, for instance at larger agrivoltaic facilities, at different agrivoltaic systems like vertical or tracking systems, in other landscape contexts, and on different species groups and is particularly necessary for elaborating recommendations on design, operation, and management of agrivoltaic facilities.

## Author Contributions

**Lara Diekmann:** conceptualization (equal), data curation (lead), formal analysis (lead), funding acquisition (equal), investigation (equal), methodology (equal), project administration (supporting), visualization (lead), writing – original draft (lead), writing – review and editing (equal). **Felix Zitzmann:** formal analysis (supporting), investigation (equal), methodology (supporting), writing – review and editing (equal). **Frank Schaarschmidt:** formal analysis (supporting), methodology (equal). **Michael Reich:** conceptualization (equal), funding acquisition (equal), methodology (equal), project administration (lead), writing – review and editing (supporting).

## Conflicts of Interest

The authors declare no conflicts of interest.

## Data Availability

Data is available on Research Data Repository of LUH (Leibniz University Hannover): https://doi.org/10.25835/9lpk4egz.

## Appendix A

**TABLE A1:** Recorded bird species in the study area and within the five plots (av = agrivoltaic, ct = control, e1 = established I, e2 = established II, tr = tree row), sorted by their frequency of detection (recorded through survey by standardized counts and/or detection by camera traps) during the investigation period no. on av and ct: Days with survey by standardized counts (*n* = 27 days)/days with detection by camera traps (*n* = 363 days); no. on e1, e2, and tv: Days with survey by point counts (*n* = 27).

Species name		RL^a^		No. of days with record on the plots				
		G	L	av	ct	e1	e2	tr
White Wagtail	*Motacilla alba*	*	*	18/121	10/90	9	10	5
Common Linnet	*Linaria cannabina*	!	!	2/35	1/39	1	5	5
Yellow Wagtail	*Motacilla flava*	*	*	4/14	4/31	7	8	6
Wood Pigeon	*Columba palumbus*	*	*	3/20	3/29			3
Black Redstart	*Phoenicurus ochruros*	*	*	8/21	2/8			3
Yellowhammer	*Emberiza citrinella*	*	N	2/10	3/1	4	1	10
Tree Sparrow	*Passer montanus*	N	N	4/6	2/0	3	8	6
Woodlark	*Lullula arborea*	N	N	0/9	0/12			
Common Chaffinch	*Fringilla coelebs*	*	*	1/4	0/4	2	3	9
Common Starling	*Sturnus vulgaris*	!	!	1/8	0/6		3	3
Great Tit	*Parus major*	*	*	5/0				12
Common Blackbird	*Turdus merula*	*	*	1/6			1	3
Eurasian Skylark	*Alauda arvensis*	!	!		0/3	5	5	
Fieldfare	*Turdus pilaris*	*	*	0/1			6	9
Gray Partridge	*Perdix perdix*	!!	!!	0/6	1/2		1	
Common Buzzard	*Buteo buteo*	*	*	2/0			1	8
Barn Swallow	*Hirundo rustica*	N	!	2/0	2/0	5	4	
Eurasian Blue Tit	*Cyanistes caeruleus*	*	*					8
Northern Raven	*Corvus corax*	*	*		0/4	1	1	2
Song Trush	*Turdus philomelos*	*	*	0/5	0/1		1	
Common Pheasant	*Phasianus colchicus*	x	x	0/5	0/2			
Eurasian Jay	*Garrulus glandarius*	*	*	1/0				6
Common House Martin	*Delichon urbicum*	!	!	3/0		3	2	
Red‐backed Shrike	*Lanius collurio*	*	N				2	4
Common Kestrel	*Falco tinnunculus*	*	N	1/0	1/0		1	
Eurasian Siskin	*Spinus spinus*	*	*					3
European Greenfinch	*Carduelis chloris*	*	*	0/2				1
Red Kite	*Milvus milvus*	*	!			1	2	
Rook	*Corvus frugilegus*	*	*	0/1	0/3			
Carrion Crow	*Corvus corone*	*	*	0/2				
Eurasian Golden Oriole	*Oriolus oriolus*	N	!					2
European Robin	*Erithacus rubecula*	*	*					2
Great Egret	*Casmerodius albus*	x	x				2	
Gray Heron	*Ardea cinerea*	*	N		1/0		1	
Common Chiffchaff	*Phylloscopus collybita*	*	*					1
Common Nightingale	*Luscinia megarhynchos*	*	N	0/1				
Common Swift	*Apus apus*	*	*			1		
Dunnock	*Prunella modularis*	*	*					1
Egyptian Goose	*Alopochen aegyptiaca*	x	x	0/1				
Eurasian Collared Dove	*Streptopelia decaocto*	*	*					1
European Goldfinch	*Carduelis carduelis*	*	N		0/1			
Green Sandpiper	*Tringa ochropus*	*	*	1/0				
Mistle Trush	*Turdus viscivorus*	*	*		0/1			
Short‐toed Treecreeper	*Certhia brachydactyla*	*	*					1
Willow Warbler	*Phylloscopus trochilus*	*	*					1
No. of species per plot				17/21	11/17	12	21	26
No. of observations by bird surveys				156	62	146	443	368
No. of detections by CTs (*n* = 1291)				775	516	—	—	—
Total no. of species				45				

^a^ RL = Red List Status for Breeding Birds in G = Germany (Ryslavy et al. 2020) and L = Lower Saxony (Krüger and Sandkühler 2022); Categories: * = Least Concern, N = Near Threatened, ! = Vulnerable, !! = Endangered, x = no list entry.
